# European Turtle Dove Population Trend in Greece Using Hunting Statistics of the Past 16-Year Period as Indices

**DOI:** 10.3390/ani12030368

**Published:** 2022-02-03

**Authors:** Christos Thomaidis, Konstantinos G. Papaspyropoulos, Theophanis Karabatzakis, George Logothetis, Gesthimani Christophoridou

**Affiliations:** 1 General Department/Department of Forestry and Natural Environment Management, Agricultural University of Athens, 36100 Karpenisi, Greece; 2 ARTEMIS Scientific Team, 66100 Drama, Greece; fanis_karampatsakis@yahoo.gr (T.K.); logothetis.g@gmail.com (G.L.); gxristof@yahoo.gr (G.C.); 3 Laboratory of Forest Economics, Department of Forestry and Natural Environment, Aristotle University of Thessaloniki, 54124 Thessaloniki, Greece; kodafype@for.auth.gr

**Keywords:** European turtle dove, harvest characteristics, hunting sustainability, population status, state-space models

## Abstract

**Simple Summary:**

Hunting regulates the populations of hunted species. By collecting annual data of hunting activity, scientists may estimate the trend of the population numbers of these species. In Greece, the ARTEMIS project (named after the ancient Greek goddess Artemis (Diana)) is a statistical database of hunting characteristics, as revealed by questionnaires distributed to hunters. In the present research, these hunting statistics are used to determine the population trend of the European turtle dove in the country, an important species to Greek hunters. By using advanced statistical modeling, the research finds that for the period 2004/05–2019/20, the population trend of the European turtle dove in Greece is stable and its harvest sustainable.

**Abstract:**

The European turtle dove is an important game bird for the hunters in Greece, which is one of a few European countries where its hunting is allowed. The sustainability of the species’ hunting in Europe is discussed during the last several years due to declines in its population, which forced IUCN to classify it as vulnerable. In Greece, its harvest takes place from 20 August and lasts as long as the presence of the species in the country (mid-October). The ARTEMIS project is a Greek statistical database of hunting characteristics, as revealed by questionnaires distributed to hunters. Statistical indicators such as hunting opportunity and hunting harvest are considered in the literature as reliable to show the population trend of a game species. Therefore, in the present research, hunting statistics are used to determine the population trend of the European turtle dove in Greece. State-space modeling was the main procedure used, a method which allows us to deal with errors that exist from hunting bag data or hunting opportunity data assuming that on average the under and overestimations will be equal. The results of the modeling analysis show a stable trend of the variables used, i.e., hunting opportunity, hunting harvest, and juveniles to adult’s ratio. Additionally, the hunting sustainability index showed that the sustainability of the species is improved annually, as a slight positive trend is revealed. This is in favor of the species, if it is considered that the actual percentage of the turtle dove population harvested is lower, since not all doves are encountered by hunters. It is concluded that for the period 2004/05–2019/20, as indicated by the hunting statistics, the population trend of the European turtle dove in Greece was stable and its harvest sustainable.

## 1. Introduction

The European turtle dove (*Streptopelia turtur*) is an important game species in Greece. The species breeds over much of the Greek mainland, mainly in the north, as well on some Aegean and Ionian Islands [1]. Greece is one of the 10 countries in Europe where the hunting of the species is allowed [2], as also confirmed by the EU Birds Directive [3].

The European turtle dove is classified as globally threatened (vulnerable) [4]. Turtle doves have declined in many parts of Europe. The decline is more pronounced in the countries of the western flyway [5]. Greece belongs to the eastern flyway [6] together with Egypt, East Europe and the Middle East [7].

The sustainability of the species hunting in Europe is discussed during the last several years due to a decline in its population [8,9,10]. In 2018, the European Commission approved the International Species Action Plan [2], which suggested a temporary hunting moratorium. In Greece and other European Union countries, this moratorium hasn’t been implemented yet [8]. However, the country has put hunting bag restrictions into force after 2018 and currently, in 2021, an online system (mobile application) is implemented where hunters report their harvest after their outings. The daily limit is six birds. Until the total quota of almost 150 thousand hunted turtle doves is reached, the hunting of the species is allowed. This limit has not yet been reached as of 23 November 2021.

In Greece, the length of the hunting season and daily bag limits for all game species are set by ministerial decision every year, signed prior to the opening of the hunting season, after taking into account all available information on the population trend and status of these species. Hunting is taking place on public land, and it is also allowed on private land, except in special cases of standing crops or fences.

The hunting season for the European turtle dove officially runs from 20 August to 28 February, but practically lasts as long as the presence of the species in the country. Hunting is allowed every day during daylight hours, with a daily bag limit of 12 birds (10 in 2018/19, 8 in 2019/21 and 6 in 2021/22) per hunter. From 20 August to 14 September, hunting is permitted only within designated areas (“Passage Zones of Migrating Birds”), covering approximately 25% of the Greek territory. After that, hunting is permitted all over the country, except in areas closed to hunting (ca.13% of territory). The use of live or artificial decoys, mouth or electronic calls, as well as any form of bait, is prohibited for all game species under the Greek Game Law.

Hunters take stands and wait for doves near feeding fields with standing or recently harvested crops (normal agricultural operations), along travel corridors between roosting, watering and feeding areas or along migration routes.

There is relatively little published information on harvest data for this species in Europe. Researchers have published on turtle dove numbers bagged annually for certain years in France, refs. [11,12,13,14] authored a report containing harvest information on this species for Spain. Reviews of available data of hunting bag statistics for the 10 EU countries who practice turtle dove hunting was presented by [2,15,16,17].

Harvest data are a valuable source of information for game managers and serve as a useful index of population trends of game species [18,19,20,21,22,23]. These data are usually collected systematically for long periods of time with a stable methodology, and sometimes they are the only available data for estimating population trends for wildlife species [24].

The number of game animals encountered per unit time is considered a valid index of relative population abundance by a number of researchers, expressed as “abundance”, “hunting success” or “hunting opportunity” (flushes of individual birds per hour of hunting—[25,26] coveys per hour of hunting—[27,28]), “hunting index of abundance” (number of birds seen per hunting trip [20,29] or “numbers seen per hour” [30].

Age ratios of bagged animals have been used rather widely as an index of annual production of young in game populations. They may not be the true ratios in the population, but by comparing annual values over several years, a trend of breeding success can be assessed [25,31,32,33,34,35].

Therefore, the aim of the research is to contribute to the debate taking place for the hunting of the species by analyzing harvest data of the European turtle dove in Greece. This way, the population trend of this species in Greece is assessed and the sustainability of its hunting in the country is discussed.

## 2. Materials and Methods

Harvest data for turtle doves were collected through Project “ARTEMIS: Recording harvest parameters in Greece and monitoring game species population dynamics”. It is run and financed by the Hellenic Hunters Confederation.

The project, named after the ancient Greek goddess Artemis (Diana), has two legs: ARTEMIS I for recording the harvest data and ARTEMIS II for collecting biological material (parts) to determine the age structure of selected game species from harvested samples, as an index of reproductive output.

Harvest data are collected through hunters who complete a statistical questionnaire with the daily results of all their hunting outings throughout the hunting season. The questionnaire works like an annual notebook diary for the hunter who records hunting statistics after every outing. In total, 30 thousand printed questionnaires with postage paid return envelops are distributed in random to hunters annually through all the 252 local hunting associations, upon renewal or first issuance of their license.

Variables recorded for each outing are game species, date, location of hunt (prefecture), number of hunters in the party, number of heads of game encountered within shooting range (“hunting opportunity”), number of heads of game shot and retrieved (“hunting harvest”) and duration (measured in hours, at half hour increments). Participation of hunters is voluntary. At the end of the hunting season, hunters independently return their completed questionnaire to the ARTEMIS Scientific Team for data analysis.

Data for the hunting seasons 2004/05 and 2019/20 are presented for ARTEMIS I. Questionnaires collected ranged from 927 to 1132 annually representing approximately 0.55–0.64% of the licensed hunters, belonging to 180–206 out of a total of 252 local hunting associations. A smaller proportion was the turtle dove hunters who were defined as individuals who have hunted at least one turtle dove during all their annual outings, a definition also used in national surveys in France [36,37].

The statistical unit is the participating hunter. Mean annual hunting opportunity (turtle doves encountered) per hour per hunter is used as indicator of population abundance, since they are independent of the duration of hunting outing.

Total annual harvest is not used as an abundance indicator because it depends on personal factors, hunter effort and number of licensed hunters, which may change across time.

The hunting sustainability index is calculated as 1-mha/h/mop/h, where m ha/h is the mean annual harvest per hour and mop/h is the mean annual hunting opportunity per hour, per hunter.

Turtle dove wings were collected by the Game Warden Body of the seven (7) Hellenic Hunting Federations. This Body is funded solely by the hunters through their annual license fee. Upon checking hunters in the field, game wardens collect from each hunter the right wing from one or more of the doves harvested, as the hunter allows. Each wing is tagged with a numbered tag, bearing the date and location of harvest and placed in a plastic bag. Wings are then frozen and stored until the end of the hunting season. The specimens collected are mailed to the ARTEMIS Scientific Team office, where they are thawed, dried and identified as to the age (juvenile < than 1 year and adult > 1 year old).

A total of 7664 wings were collected for the period 2006/07 and 2019/20 for ARTEMIS II. Harvest data are communicated annually to the Ministry of Environment by the Hunters’ Confederation.

We used state-space modelling to model the univariate time series of (a) mean annual hunting opportunity and (b) mean annual hunting harvest, per hour per hunter, (c) hunting sustainability index, and (d) ratio of juveniles to adults. State space models are appropriate for correcting the errors that take place from the observations of the data collectors. In fact, they may reveal the unobserved demographic processes from the collected observation data [38].

In general, state-space models allow us to deal with errors that exist from hunting bag data or hunting opportunity data assuming that on average the under- and overestimations will be equal [38]. In the case of hunting harvest, for example, the state-space model corrects inappropriate harvest limits, or weather conditions that dislocate birds from the area that the hunters expect to find them in.

We used the methodology described in [39] for the modelling. A state-space model is described by two equations. One for the data y and one for the hidden state x, as follows:

x_t_ = bx_t−1_ + w_t_ where w_t_~N(0,q) (1)

y_t_ = x _t_ + v_t_ where v_t_~N(0,r) (2)

x_0_ = μ (3)

where y is the data and x is a hidden random walk estimated from the data [39], b the coefficient of x and w_t_ and v_t_ normally distributed errors.

We modeled (a) hunting opportunity per hour per hunter, (b) hunting harvest per hour per hunter, (c) hunting sustainability time series for a 16-year time span (2004/05–2019/20) and (d) percentage of juveniles to adults for a 14-year time span (2006/07–2019/2020) (Table S1).

For each of the four-time series we fitted three different models to the data.

The three models are:

1. The flat level model: in this case, the data are fitted as a simple average with variability around some level μ. Thus, this model reveals a stable population status in the time span.
2. The linear trend model: in this case, the data are fitted accounting for an average per-year rise or decline. Such a model can reveal decline or increase in the population trend.
3. The stochastic level model: this model allows the mean of the fitted variable to change and estimates sudden changes in the data. This is an autoregressive process where the average of the data in year t is a function of the average in year t-1 [39].

We compared the models with the Akaike Information Criterion (AIC). Due to the short time series of the four variables, we used the AICb version. AIC and AICc tend to choose complex models in such time series; therefore, [40] propose an algorithm with innovations bootstrap to correct this error [39]. The AICb is estimated with 1000 bootstraps. We also checked the model residuals to test if the best model fits well to the data.

The data were analyzed in R using the package MARSS (Multivariate Auto-Regressive (1) State-Space) in its 3.11.3 version. The MARRS package is suitable for estimating the parameters of linear MARSS models with Gaussian errors [41].

## 3. Results

### 3.1. Descriptive Statistics

A total of 33,884 hunting outings were recorded for turtle doves by individual hunters participating in the ARTEMIS during the 16-year period. Outings for turtle doves represented 4.94%–6.95% of the total outings reported annually for all game species. Despite the long official hunting season, turtle doves are actually exposed to hunting for less than 2 months.

First peak in abundance, as indicated by hunting opportunity, was recorded during the first week (20–26 August) of the season. In the year 2004–2005, the first peak was during the second week (27 August–2 September), as the hunting season opened a week later that year. Breeding birds and young of the year are still present in Greece at this time.

**Figure 1:**
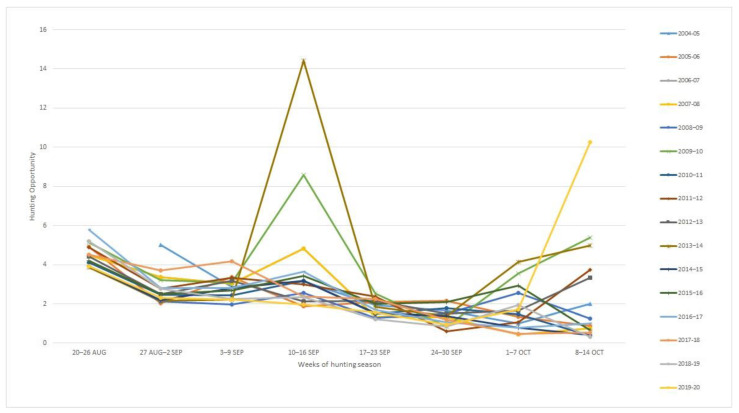
Evolution of mean annual hunting opportunity per hour per hunter for turtle doves during the hunting seasons 2004/05 and 2019/20 in Greece.

The second peak was during the fourth week (10–16 September), when the main autumn passage takes place and numbers are augmented by turtle doves from other countries. Turtle doves are passing through up to the middle of October, some years in fair numbers (Figure 1).

**Figure 2:**
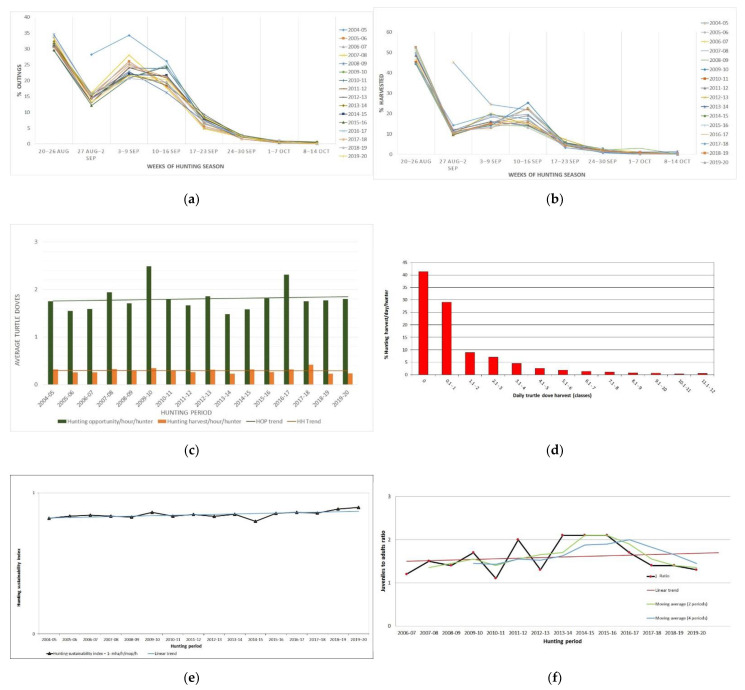
Hunting statistics during the hunting seasons 2004/05 and 2019/20 in Greece: (**a**) Percent distribution of cumulative hunting demand for turtle doves; (**b**) Percent distribution of cumulative hunting harvest for turtle doves; (**c**) Evolution of mean annual hunting opportunity and harvest per hour per hunter for turtle doves; (**d**) Cumulative percent distribution of daily turtle dove harvest per hunter; (**e**) Evolution of the hunting sustainability index of the turtle dove; (**f**) Evolution of the juvenile to adult ratio from wings of harvested turtle doves.

Hunting demand, expressed as a number of outings for turtle doves, was at its highest during the first week of the season (29.52–34.52%). Demand during the second week dropped considerably (12.14–15.86%). The exception was the year 2004–2005 with 28.20% demand, due to the aforementioned reason. Demand rose again during the 3rd and 4th week (20.69–34.26% and 16.23–26.09%, respectively), due to the hunters’ anticipation of migratory movements of the birds and fell sharply thereafter (Figure 2a)

The largest proportion of annual cumulative harvest for this species was recorded during the first week of the hunting season (44.02–52.44%). During the first three weeks, harvest reached 68.76–79.37% of the total annual figure. On the 4th week, despite high availability of doves due to migration (main passage), only 12.93–25.27% of the annual harvest was recorded (Figure 2b).

Mean annual hunting opportunity per hour per hunter ranged between 1.48 and 2.49 birds with a mean value of 1.80 (SE = 0.066). The mean annual harvest per hour per hunter ranged between 0.23 and 0.41 birds with a mean value of 0.29 (SE = 0.0126) (Figure 2c).

Mean annual number of hunting outings per hunter ranged between 4.16 and 5.27 (mean = 4.86, SE = 0.062).

The percentage of hunters that have bagged at least one turtle dove per season reached 34.19–43.58% of participants in the ARTEMIS I Program. Mean annual harvest per hunter was 4.44 birds (range 4.03–5.58, SΕ = 0.104). Distribution of daily harvest shows that 41.34% of hunters were unsuccessful, 23.12% bagged 1.1–5 birds per outing and only 1.84% or less of hunters reached middle or upper values of the bag limit (Figure 2d).

The hunting sustainability index ranged between 0.7984 and 0.8971 with a mean value of 0.8458 (SE = 0.006), meaning that 79.84–89.71% of the doves encountered by a hunter per hour evaded harvest and only 10.29–20.16% fell to the gun (Figure 2e).

The ratio of juveniles to adults ranged between 1.20:1 and 2.11:1 with a mean value of 1.59:1 (SE = 0.0954), which means that the juveniles were always more numerous than the adults (Figure 2f).

### 3.2. State-Space Modeling

#### 3.2.1. Mean Annual Hunting Opportunity Per Hour Per Hunter

Τhe model with the smallest AICb is the flat level model. This is considered the best of the three models, as shown in Table 1. The analysis of the model residuals show that they are fluctuating around zero. Hunting opportunity during the 16-year period fluctuates around a mean value, confirming that it is stable.

#### 3.2.2. Mean Annual Hunting Harvest Per Hour Per Hunter

The best model seems to be the flat model as AICb is the minimum in this case, which is presented in Table 2. The residuals are distributed around zero and thus the model fits well to the data. Therefore, the hunting harvest series for the last 16 years is stable.

#### 3.2.3. Hunting Sustainability

The hunting sustainability index was best described, as shown in Table 3, by the linear trend model. AICb is smaller than the other two models. The trend u may be small, but it is positive with a positive 95% confidence interval. The trend shows that hunting sustainability index is increased by a quantity of 0.003069 every year, with a mean value of 0.819802, as estimated by the model. Accordingly, only an average 18.02% of the birds encountered by hunters are harvested, decreased by a factor of 0.3%, every year.

#### 3.2.4. Juvenile to Adult Ratio

Table 4 shows that the flat level model seems to describe the data best (it has the smallest AICb); thus, the time series is stable for the 14-year period indicated. As estimated by the model, on average the juveniles are annually 20% more than the adults in the population.

## 4. Discussion

The pattern of mean annual hunting opportunity indicated the presence of an increased number of turtle doves during the first week of the season (20–26 August), since there is little migration activity in Greece at this time.

No migratory activity of turtle doves in August is reported by [42], the earliest date being 2 September. The main passage took place from 9 September through the 15 September. The latest date was 29 September, while the ARTEMIS data show that turtle doves pass through till the middle of October. According to [6], high migration activity for the Eastern Flyway (at 40 o latitude, the approximate latitude for Greece) takes place in September, with smaller passages in October and even November and December.

Harvest was at its highest during the first week of the hunting season, so the local population is mostly affected. The exception was the year 2004/05 when hunting started a week later. Harvest percentage rose again during the main autumn passage, but it was almost half of that of the first week, despite increased hunting opportunity. Hunters at traditional stop-over sites, such as islands and southern peninsulas are mostly active at this time. Thus, there is no heavy hunting pressure on turtle doves migrating through Greece.

Mean annual outings per hunter were few, probably due to the short duration of the season (presence of the species in Greece) and the fact that the largest proportion of hunting harvest takes place during the first week. It may also be caused by a shift of interest towards other game species whose season coincides with turtle dove dates such as hare and wild boar (opening on 15 September) or other migratory birds, such as thrushes, woodcock, and woodpigeon, especially during October, when numbers of turtle doves are dropping [43].

Mean annual harvest per hunter also retained low values for the aforementioned reasons. Mean annual harvest of turtle doves in France is lower, ranging between 2.5–3.6 birds per hunter, depending on the reporting source [12]. Furthermore, the distribution of daily harvest indicates that it is maintained at low levels.

The mean annual hunting opportunity and the hunting harvest per hour per hunter time series for 16 years show that the flat model best describes these data and that they fluctuate around a stable value over time.

Hunting opportunity as an indicator of population abundance shows the stability of the turtle dove population in Greece. Hunting harvest constantly retained low values compared to hunting opportunity; thus, is a good indicator of sustainable harvest and stability of the turtle dove population. A stable trend of this species is reported in Greece for the years 2007–2010 and an increasing trend for the period 2011–2014 based on the European Breeding Bird Atlas 2 [44]. Similar findings are reported in [2], and [45] who have found that both the short- and the long-term population trend of the species is stable in Greece. A recent study for 246 breeding birds in Greece finds an increasing trend for the species in Greece, similar to our finding, as indicated by the hunting sustainability index [46].

The hunting sustainability index showed that the sustainability of the species is improved annually, as a positive linear trend is revealed by the data. The hunting sustainability index shows that the ability of harvesting turtle doves under given conditions and regulations in Greece is finite, adapting to the available population levels. The actual percentage of the turtle dove population harvested is lower than that indicated by the sustainability index, since not all doves are encountered by hunters.

The juvenile to adult ratio of hunted turtle doves is another positive indicator of the species sustainability in Greece, fluctuating around a mean of 1.59:1 and showing a stable value of 20%, on the average, more juveniles during the 14-year period. Comparatively, [47] reported an age ratio of 1.4:1 young per adult turtle doves from bag records in Spain, while [48] reported mean ratios 1.49:1 and 0.76:1 of juveniles to adults for harvested turtle doves in 1997 and 1998, respectively, from seven areas (zones) in Andalusia, Spain. Mean ratios of 0.50:1–0.77:1 juveniles to adults from three Spanish provinces are given by [14], and 1.69:1 ratio from a fourth one, for hunted turtle doves, for the year 2012. Finally, [49] reported a mean age ratio of 1.84:1 juveniles to adults of hunted turtle doves from 20 hunting estates in Extremadura, Spain, offering supplemental food, while at 20 other estates not offering supplemental food, the ratio was 1.38:1 for the year 2009.

Future research for the population trend of the European turtle dove in Greece should focus on testing whether a full random, compared to volunteer-based, sample produces the same or different results for the sustainability of hunting. The randomness of the sample is considered as minimizing the selection bias produced when hunters voluntarily participate in the harvest data surveys [50]. However, as stated in [50] “…If one considers that the hunting bags of a game species can serve as an index of abundance for that species (e.g., the case of wild boar Sus scrofa in the ENETWILD project), then, for relative abundance assessment, in practice we only need that the self-selection bias of hunting bag estimates is held relatively constant in time and space…”. It is expected that the timeless operation of the ARTEMIS project covers the prerequisites set by these researchers.

## 5. Conclusions

Hunting opportunity as an indicator of population abundance shows the stability of the turtle dove population in Greece. Stable trends of hunting opportunity and harvest are good indicators of hunting sustainability.

Constant improvement of the hunting sustainability index throughout the 16-year period further supports hunting sustainability of turtle doves in Greece.

The above positive indicators, as well as the stability of juvenile to adult time series, attest the stability of population trends of this species in the country.

## Figures and Tables

**Table 1:** Comparison of the three models for the hunting opportunity variable.

Model	Parameter	Estimation	Std Error	95% CI	AICb
Flat	μ	1.8016	0.064	1.6761, 1.927	7.898878
	sd	0.0656	0.0232	0.0201, 0.1110	
Linear trend	μ	1.75188	0.1335	1.4904, 2.0135	10.61884
	sd	0.06479	0.0229	0.0199, 0.1097	
	u	0.00589	0.0138	−0.0212, 0.0329	
Stochastic	μ	1.8016	0.06534	1.67350, 1.92962	9.445684
	sd	0.0656	0.02797	0.01075, 0.12038	
	q	0.000	0.00184	−0.00361, 0.00361	

**Table 2:** Comparison of the three models for the hunting harvest variable.

Model	Parameter	Estimation	Std Error	95% CI	AICb
Flat	μ	0.29033	0.012231	0.266358, 0.31430	−45.63137
	sd	0.00239	0.000846	0.000735, 0.00405	
Linear trend	μ	0.294781	0.025623	0.244560, 0.34500	−41.71858
	sd	0.002387	0.000844	0.000733, 0.00404	
	u	−0.000528	0.002650	−0.005722, 0.00467	
Stochastic	μ	0.29033	0.00127	0.265442, 0.315221	−42.78692
	sd	0.00239	0.000976	0.00048, 0.004307	
	q	0.0000	0.0000572	−0.000112, 0.000112	

**Table 3:** Comparison of the three models for the hunting sustainability variable.

Model	Parameter	Estimation	Std Error	95% CI	AICb
Flat	μ	0.845792	0.00579	0.834435, 0.85715	−69.53039
	sd	0.000537	0.00019	0.000165, 0.00091	
Linear trend	μ	0.819802	0.009557	0.801071, 0.838533	−73.58447
	sd	0.000332	0.000117	0.000102, 0.000562	
	u	0.003069	0.000988	0.001132, 0.005006	
Stochastic	μ	0.827625	0.015187	0.798, 0.857391	−46.87441
	sd	0.000222	0.000144	−0.0000617, 0.000505	
	q	0.000118	0.0000432	−0.000131, 0.000367	

**Table 4:** Comparison of the three models for the juvenile to adult ratio variable.

Model	Parameter	Estimation	Std Error	95% CI	AICb
Flat	μ	1.587	0.0919	1.4067, 1.767	15.79941
	sd	0.118	0.0447	0.0307, 0.2060	
Linear trend	μ	1.4872	0.1917	1.1115, 1.8628	19.25895
	sd	0.1153	0.0436	0.0299, 0.2007	
	u	0.0134	0.0225	−0.0307, 0.0575	
Stochastic	μ	1.587	0.10349	1.38405, 1.78971	16.93824
	sd	0.118	0.04747	0.02533, 0.21140	
	q	0.0000	0.00212	−0.00415, 0.00415	

## Data Availability

The data presented in this study are available online at https://www.mdpi.com/article/10.3390/ani12030368/s1, Table S1: Time series of analyzed variables.

## Supplementary Materials

The following supporting information can be downloaded at: https://www.mdpi.com/article/10.3390/ani12030368/s1, Table S1: Time series of analyzed variables.
